# Tuning of mRNA stability through altering 3′-UTR sequences generates distinct output expression in a synthetic circuit driven by p53 oscillations

**DOI:** 10.1038/s41598-019-42509-y

**Published:** 2019-04-12

**Authors:** Woo Seuk Koh, Joshua R. Porter, Eric Batchelor

**Affiliations:** Laboratory of Pathology, Center for Cancer Research, National Cancer Institute, National Institutes of Health, Bethesda, MD 20892 USA

## Abstract

Synthetic biological circuits that can generate outputs with distinct expression dynamics are useful for a variety of biomedical and industrial applications. We present a method to control output dynamics by altering output mRNA decay rates. Using oscillatory expression of the transcription factor p53 as the circuit regulator, we use two approaches for controlling target gene transcript degradation rates based on the output gene’s 3′-untranslated region (3′-UTR): introduction of copies of destabilizing AU-rich elements into the 3′-UTR or swapping in naturally occurring 3′-UTRs conferring different transcript stabilities. As a proof of principle, we apply both methods to control the expression dynamics of a fluorescent protein and visualize the circuit output dynamics in single living cells. We then use the naturally occurring 3′-UTR approach to restore apoptosis in a tunable manner in a cancer cell line deficient for caspase-3 expression. Our method can be readily adapted to regulate multiple outputs each with different expression dynamics under the control of a single naturally occurring or synthetically constructed biological oscillator.

## Introduction

Biological circuits in which a single input differentially regulates multiple outputs are beneficial in both naturally occurring and synthetically constructed contexts as they enable a common stimulus to drive multiple downstream effects. Often distinct output pathways must be activated at distinct times or with distinct expression levels to generate desired cellular responses. Using a biological oscillator as an input regulator may be a potentially useful mechanism for generating such temporal control in multi-output circuits.

Recent work has shown that transcription factors with oscillatory expression dynamics can generate multiple qualitatively distinct temporal expression patterns for the genes they regulate^1–5^. For example, the transcription factor p53, whose protein levels oscillate in response to DNA double strand breaks^6–8^, has target genes with a spectrum of temporal expression patterns^2,3,9,10^. The key biological parameter that dictates specific expression dynamics of the target genes is the mRNA decay rate of each target, with rapidly decaying targets having oscillatory mRNA expression at one extreme and stable targets having mRNA levels that monotonically increase in time at the other extreme^2,3^. The mRNA expression dynamics for targets can be altered by changing the frequency of p53 oscillations, especially for those genes with mRNA decay rates near the normal p53 oscillation frequency^2^.

Engineering the stability of target gene mRNAs is a potential alternative strategy for generating specific mRNA expression dynamics of genes regulated by an oscillating transcription factor. Several factors affect mRNA stability in eukaryotic cells, including mRNAses and other RNA-binding proteins^11^. To alter the stability of a transcript encoding a protein product of interest, manipulation of noncoding regions is desirable to maintain polypeptide sequence and thus protein identity. mRNA stability can be affected by sequences encoded in the 3′-untranslated region (3′-UTR) of eukaryotic transcripts^12–14^. For example, regulatory sequences in the 3′-UTR containing adenylate-uridylate-rich elements (AU-rich elements; AREs) can destabilize mRNAs^12–14^. Thus, due to their placement in noncoding regions as well as their relatively short sequence lengths, 3′-UTR AREs provide a potentially useful strategy for altering mRNA expression dynamics in a controlled manner.

In this study, we show that synthetic target expression dynamics in response to p53 oscillations can be regulated through directed perturbation of mRNA stability. We perturb target mRNA stability through two methods: using naturally occurring 3′-UTRs of different stabilities engineered to novel cDNAs; and destabilizing an mRNA by introducing varying copies of AREs into an engineered 3′-UTR. As a proof of principle, we first applied both strategies to alter the expression dynamics of a fluorescent protein reporter under the control of a p53-responsive synthetic promoter induced by DNA double strand breaks. We then used *CASP3* (encoding the pro-apoptotic factor caspase-3) as the target gene with a variety of 3′-UTRs to restore apoptosis in a regulated manner in the caspase-3-deficient breast carcinoma cell line MCF-7. Our study highlights the potential of using an oscillatory biological circuit to generate desired target gene expression dynamics for bioengineering and translational medicine applications.

## Results

### Altering output transcript stability with AU-rich elements generates distinct expression dynamics from a biological oscillator

**Figure 1:**
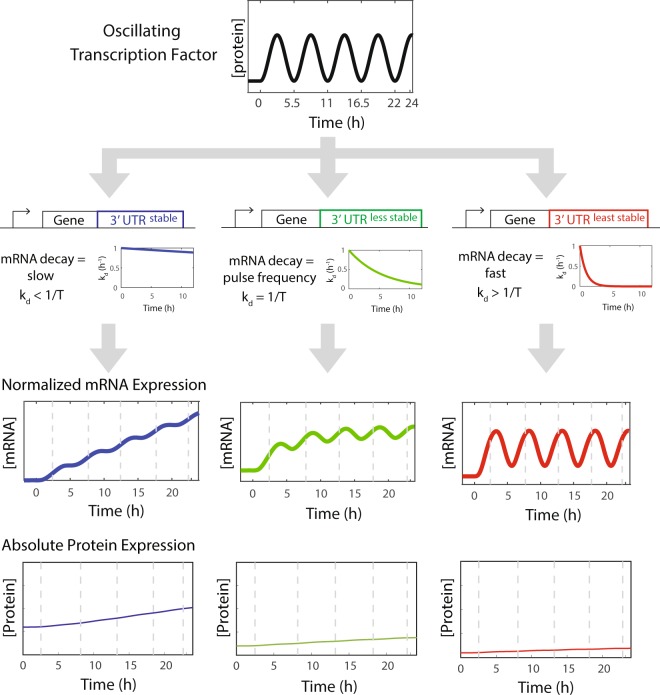
An oscillating transcription factor generates distinct output expression dynamics as a function of mRNA decay rates. A model predicts that distinct mRNA and protein expression dynamics can be generated by altering the transcript stability of target genes induced by an oscillating transcription factor. 3′-UTRs can confer different transcript stabilities. Based on the relationship between the transcript decay rate and the oscillator frequency, output gene transcripts will have rising, weakly pulsing, or strongly pulsing mRNA expression dynamics, leading to alterations in the rate of accumulation of an output protein product.

For genes induced by a transcription factor with oscillatory expression dynamics, mRNA expression can exhibit a range of dynamics from oscillatory to continuously rising (Fig. 1; ref.^2^). The decay rate of the target mRNA relative to the frequency of the transcription factor oscillations is a key factor in determining the dynamics (Fig. 1). 3′-UTRs can confer different decay rates to mRNAs, and therefore we predicted that the expression dynamics of a long-lived protein encoded by mRNAs with distinct 3′-UTRs would show a range of absolute levels of expression and rates of accumulation (Fig. 1).

**Figure 2:**
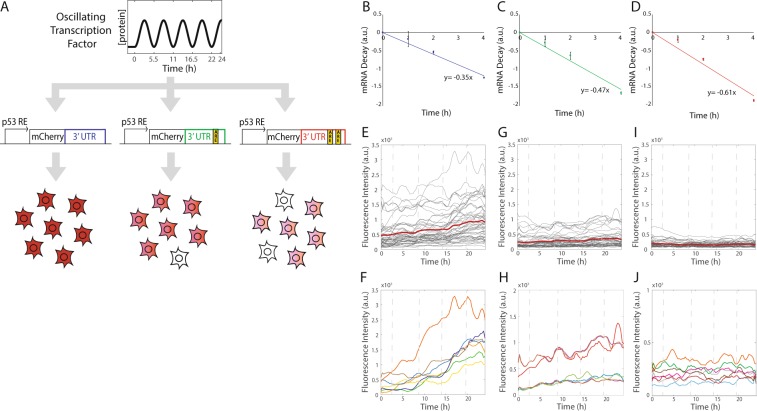
AU-rich elements destabilize output transcript stability to generate distinct expression dynamics of mCherry expression induced by p53 oscillations. (**A**) Schematic of the synthetic circuit with output reporter constructs with 0,1, or 2 AREs inserted into the *TP53I3* 3′-UTR following the *mCherry* coding sequence. Predicted phenotype for output mCherry expression in individual cells. (**B**–**D**) mRNA decay rates of *mCherry* transcripts containing 0 (**B**), 1 (**C**), or 2 (**D**) ARE sequences. All data are normalized by *GAPDH* transcript levels. Data are mean values ± SEM for 5 biological replicates. (**E**-**J**) Mean mCherry expression in single cells following NCS treatment to induce p53 oscillations in cells containing 0 (**E**,**F**), 1 (**G**,**H**), or 2 (**I**,**J**) AREs in the *mCherry* output transcript 3′-UTR. Gray lines represent mean nuclear mCherry expression in individual cells, red lines represent mean of the population for all cells in each condition (**E**,**G**,**I**). Representative single cell traces are shown in (**F**,**H**,**J**). Approximately 50 cells were quantified per condition.

We tested this prediction using the p53 oscillatory response to DNA double strand breaks as the input for several synthetic gene circuits. As a proof of principle, we first used a gene encoding the red fluorescent protein mCherry as the circuit output, as its dynamics can be readily visualized in single, live cells over time enabling precise quantification of expression dynamics (Fig. 2A). We constructed synthetic promoter reporter output plasmids for the p53 oscillator by expressing the cDNA encoding mCherry under the control of a p53-responsive reduced *MDM2* promoter. Downstream of the mCherry cDNA, we inserted the sequence for the *TP53I3* 3′-UTR. The *TP53I3* transcript is relatively long-lived compared with other p53 target gene transcripts, and its 3′-UTR has a relatively short sequence, making it a suitable candidate as a synthetic circuit component. We made analogous circuit output reporter constructs by altering the 3′-UTR through the addition of one or two copies of the ARE sequence 5′-TTATTTATTTATTATTTATTTATT-3′ (Fig. 2A) from the mouse *Tnf* 3′-UTR. Transfecting the plasmids individually into an MCF-7 p53-Venus clonal cell line previously characterized as expressing p53 oscillations in individual cells^7^, we developed three distinct stable MCF-7 cell lines expressing the mCherry output reporters.

To directly determine the efficacy of AREs in destabilizing the mCherry transcript, we induced expression of mCherry mRNA with the radiomimetic drug neocarzinostatin (NCS; ref.^15^) to generate DNA double strand breaks (DSBs). Simultaneously, we supplemented cell culture medium with 5-ethynyl uridine (EU) to pulse label newly synthesized transcripts for 3 h and then harvested cells upon chasing with fresh growth medium without EU^16^. We observed that the addition of ARE sequences increased the decay rate of the synthetic output transcripts, as predicted (Fig. 2B–D).

We next determined if the addition of destabilizing sequences to the 3′-UTR of the output gene could alter the protein expression dynamics for the synthetic circuit output. mCherry expression was monitored in single live cells via long-term time-lapse fluorescence microscopy for 24 h following activation of the p53 oscillator with NCS. Cells expressing the mCherry output mRNA without AREs, and therefore with the most stable transcript, showed overall higher basal expression, greater dynamic range of activation, and more step-like or rising mCherry protein expression (Fig. 2E,F). These expression dynamics were comparable to those for endogenous p53 target genes such as *TP53I3* previously characterized as having “rising” expression dynamics^2^. The addition of one or two AREs to the mCherry-*TP53I3* 3′-UTR resulted in decreases in the basal expression, dynamic range, and rate of accumulation of mCherry (Fig. 2G–J), as predicted (Fig. 1).

### Using 3′-UTRs of endogenous p53 target genes with differing stability generates distinct expression dynamics for a synthetic p53 target gene

**Figure 3:**
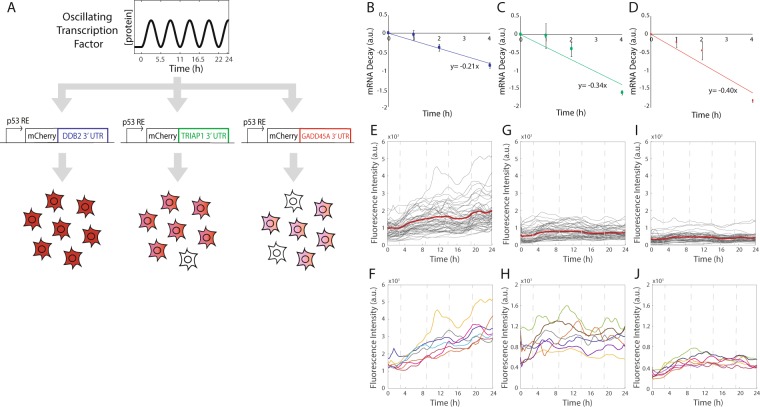
Distinct naturally occurring 3′-UTRs confer different output transcript stabilities to generate different expression dynamics of mCherry expression induced by p53 oscillations. (**A**) Schematic of the synthetic circuit with output reporter constructs with *DDB2*, *TRIAP1* or *GADD45A* 3′-UTR inserted following the *mCherry* coding sequence. Predicted phenotype for output mCherry expression in individual cells. (**B**–**D**) mRNA decay rates of *mCherry* transcripts with the *DDB2* (**B**), *TRIAP1* (**C**), or *GADD45A* (**D**) 3′-UTR. All data are normalized by *GAPDH* transcript levels. Data are mean values ± SEM for 5 biological replicates. (**E**–**J**) mCherry expression in single cells following NCS treatment to induce p53 oscillations in cells containing *DDB2* (**E**,**F**), *TRIAP1* (**G**,**H**), or *GADD45A* (**I**,**J**) 3′-UTR for the *mCherry* output transcript. Gray lines represent mean nuclear mCherry expression in individual cells, red lines represent mean of the population for all cells in each condition (**E**,**G**,**I**). Representative single cell traces are shown in (**F**,**H**,**J**). Approximately 50 cells were quantified per condition.

As a second approach to tune the expression dynamics of our synthetic output gene, we made use of naturally occurring 3′-UTRs of genes with differing mRNA decay rates. Previously, target genes of p53 had been categorized as having rising, weakly pulsing, or strongly pulsing expression dynamics, correlating with decreasing mRNA stability, respectively^2^. We selected p53 target genes from each of these three categories that had relatively short 3′-UTRs – the rising gene *DDB2*, the weakly pulsing gene *TRIAP1*, and the strongly pulsing gene *GADD45A*. Insertion of these 3′-UTR sequences downstream of the mCherry cDNA (Fig. 3A) resulted in mCherry transcripts with different decay rates (Fig. 3B–D).

We next determined the output mCherry expression dynamics for each of the p53 target 3′-UTR synthetic constructs when expression was driven by p53 oscillations. We generated three cell lines expressing the synthetic output circuits consisting of the *MDM2* reduced promoter, *mCherry* cDNA, and a p53 target gene 3′-UTR (Fig. 3A). We monitored mCherry protein expression in live cells for 24 h following induction of DNA DSBs with NCS (Fig. 3E–J). We observed that mCherry expression had the highest basal expression level, greatest dynamic range of expression, and rising dynamics for cells with the mCherry output component having the *DDB2* 3′-UTR, corresponding to the most stable transcript (Fig. 3E,F). A decrease in basal expression, dynamic range, and less characteristic rising dynamics were observed for 3′-UTRs that conferred lower stability on the mCherry output transcript (Fig. 3G–J). Comparison of integrated mCherry levels as a function of integrated p53-Venus levels in individual cells over 24 hours indicated a weak positive correlation for the *TRIAP1* (Pearson’s linear correlation ρ = 0.22) and *GADD45A* (Pearson’s linear correlation ρ = 0.25) 3′ UTR circuits, although the *DDB2* 3′UTR circuit was uncorrelated (Supplementary Fig. S1). Use of clonal cell lines genetically identical for the mCherry gene, rather than a stably transfected but genetically variable population of cells, could potentially be used to increase the correlation, if desired.

Taken together, our results provide two methods, addition of AREs and use of naturally occurring 3′-UTRs conferring distinct mRNA decay rates, for tuning the basal expression levels, dynamic range, and qualitative shape of the dynamic response for synthetic biological circuits.

### Altering transcript 3′-UTRs can restore apoptotic activity in a tunable manner

**Figure 4:**
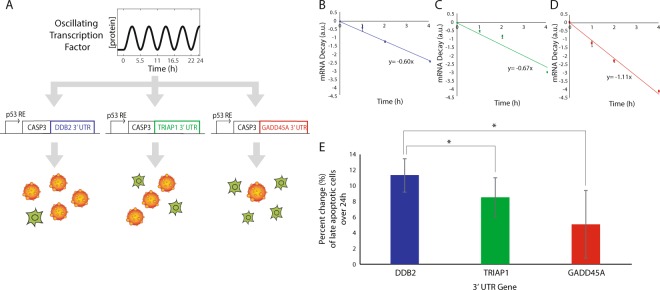
Restoration of apoptotic activity in a tunable synthetic circuit driven by p53 oscillations. (**A**) Schematic of the synthetic circuit with caspase-3 output constructs with *DDB2*, *TRIAP1* or *GADD45A* 3′-UTR inserted following the *CASP3* coding sequence. Predicted phenotype for apoptosis in individual cells. (B-D) mRNA decay rates of *CASP3* transcripts with the *DDB2* (**B**), *TRIAP1* (**C**), or *GADD45A* (**D**) 3′-UTR. All data are normalized by *GAPDH* transcript levels. Data are mean values ± SEM for 3 (**B**) or 5 (**C**,**D**) biological replicates. (**E**) Fold changes in the number of late apoptotic cells 24 h after NCS treatment for cells expression *CASP3* with different 3′-UTRs. Data represent mean ± SEM for triplicate experiments. (*indicates statistically significant difference, p < 0.05 for 1-sample ANOVA test).

Having used 3′-UTRs to alter transcript stability and circuit output dynamics in a tunable manner for a fluorescent reporter, we next developed analogous synthetic circuits with a more translationally relevant goal, restoring apoptotic activity in caspase-deficient cells. MCF-7 cells have a *CASP3* mutation, preventing effective activation of apoptosis^17^. We therefore constructed plasmids using *CASP3*, coding for the uncleaved pro-caspase 3 protein, as the circuit output gene (Fig. 4A). To the *CASP3* cDNA, we cloned 3′-UTRs corresponding to those for the p53 targets *DDB2*, *TRIAP1*, and *GADD45A* (Fig. 4A), and established stable MCF-7 cell lines expressing the plasmids. Quantification of mRNA decay rates for the *CASP3* constructs in response to DNA double strand breaks showed that addition of the different 3′-UTRs altered mRNA stability (Fig. 4B–D).

We next determined whether apoptosis could be restored in a tunable manner using the caspase-3 circuits when driven by p53 oscillations. We generated three different cell lines each with stable expression of a different *CASP3* – p53 target gene 3′-UTR output plasmid. We treated cells with NCS to induce DNA damage and initiate p53 oscillations, and we then stained cells with propidium iodide and annexin-V 24 h after NCS treatment. We observed the greatest percentage of apoptotic cells for cells expressing *CASP3* with the *DDB2* 3′-UTR (Fig. 4E), corresponding to the most stable transcripts (Fig. 4B). Fewer apoptotic cells were detected for cells expressing *CASP3* with the *TRIAP1* or *GADD45A* 3′-UTRs (Fig. 4E), corresponding to less stable transcripts (Fig. 4C,D). These results suggest that 3′-UTRs can be used to alter the stability of output mRNA transcripts, providing a tunable method to alter a variety of biological circuit dynamics.

## Discussion

Synthetic biological circuits are useful for many applications, from probing the regulation and function of naturally occurring processes to engineering completely novel metabolic pathways^18^. Since the likelihood of failure for a synthetic circuit increases as more components are added, methods for controlling multiple outputs with distinct thresholds and temporal characteristics is desirable. In this study, we present relatively straight-forward methods for generating different outputs from biological oscillators. Due to manipulating the decay rates of mRNAs through noncoding regions, our methods should be readily adaptable to a wide range of systems since they do not alter protein coding sequences.

Numerous examples of biological oscillators have been identified in natural signaling and transcriptional circuits^1,5–8,19–29^ and robust synthetic oscillators are some of the earliest examples of constructed circuits^30,31^. We previously showed that natural oscillators can drive a wide spectrum of qualitatively distinct output expression dynamics^2^. Adapting this strategy from the p53 system to generate diversity in transcriptional responses for other oscillators should be relatively simple, whether for naturally occurring oscillators such as NF-κB and circadian circuits or for synthetic oscillators such as a bacterial repressilator^30^.

The steady state levels of a transcript depend on a balance of mRNA production and degradation rates. Regulation of output transcript decay rates through alteration of 3′-UTR sequences also provides a method for tuning steady state expression levels for a wide range of synthetic transcriptional circuits. This method is effective regardless of the characteristics of the promoter driving synthesis of the transcript of interest, whether, for example, from a promoter regulated by an oscillating transcription factor, a constitutive promoter, or a promoter with a high level of burstiness^32–36^. A benefit gained from controlling the degradation rate of transcripts regulated by an oscillating transcription factor is that it provides a potential mechanism for generating a wider range of qualitatively distinct expression dynamics prior to the steady state.

There are many ways in which our initial designs might be improved to yield more optimal outputs or more complex regulatory control. For example, alternative AREs of varying numbers might enable more precise regulation of mRNA decay rates. Additionally, a larger survey of naturally occurring 3′-UTRs could potentially yield a variety of useful sequences based on size and degradation rate. Optimal choices for noncoding sequences may depend on characteristics of the specific output mRNAs for any given synthetic circuit. Finally, alteration of the protein decay rates would provide an additional level of regulation for controlling the ultimate protein expression output for many synthetic circuits. Future studies refining and building upon our methods will likely provide additional tools for the synthetic biology toolbox.

## Methods

### Plasmids

Six plasmids expressing the cDNA for the fluorescent protein mCherry, but each producing mRNA with different 3′-UTR sequences, were developed. We first extracted RNA from MCF7 cells using RNeasy (QIAGEN, 74134) and QIAshredder (QIAGEN, 79654) to collect complementary DNA samples of genes using a 20 μL reaction of High Capacity cDNA Reverse Transcription Kit (ThermoFisher, 4374966). To isolate 3′-UTRs from *DDB2*, *TRIAP1* and *GADD45A*, we performed PCR with the primers 5′-TGAGAGACACTAAAGAAGGTGTG-3′ and 5′-TTTTTTTTTTTTTTTTTTTTTTCTCAGTCGGTATGGTTTTATTG-3′ (for *DDB2*), 5′- TGACCTTGACAGTCACCTTGAAG-3′ and 5′-TTTTTTTTTTTTTTTAATGATAGAAACTAAATGTTAC-3′ (for *TRIAP1*), and 5′-TGATGGCATCTGAATGAAAATAAC-3′ and 5′-TTTTTTTTTTTTTTTTTTGAAATGATGCAATTATTCATACC-3′ (for *GADD45A*), followed by agarose gel electrophoresis and purification using a QiaQuick Gel Extraction Kit (QIAGEN, 28704). To generate Gateway cloning vectors (ThermoFisher) for the 3′-UTR sequences, we performed PCR on the purified sequences using the primers 5′-GGGGACAGCTTTCTTGTACAAAGTGGGCTGAGAGACACTAAAGAAGGTGTG-3′ and 5′-GGGGACAACTTTGTATAATAAAGTTGCTTTTTTTTTTTTTTTTTTTTTTCTCAGTCGGTATGGTTTTATTG-3′ (for *DDB2*), 5′-GGGGACAGCTTTCTTGTACAAAGTGGGCTGACCTTGACAGTCACCTTGAAG-3′ and 5′-GGGGACAACTTTGTATAATAAAGTTGCTTTTTTTTTTTTTTTAATGATAGAAACTAAATGTTAC-3′ (for *TRIAP1*), and 5′-GGGGACAGCTTTCTTGTACAAAGTGGGCTGATGGCATCTGAATGAAAATAAC-3′ and 5′-GGGGACAACTTTGTATAATAAAGTTGCTTTTTTTTTTTTTTTTTTGAAATGATGCAATTATTCATACC-3′ (for *GADD45A*), followed by agarose gel electrophoresis and purification. We also designed three different 3′-UTRs by modifying the 3′-UTR from the *TP53I3* gene (NM_004881.4). Within the *TP53I3* 3′-UTR, we inserted 0, 1, or 2 AU-rich elements (AREs; TTATTTATTTATTATTTATTTATT). The 3′-UTRs flanked by *attB2r* and *attB3* sequences for BP reaction (Gateway cloning, ThermoFisher) were obtained as gBlocks from IDT. We then performed Gateway BP reactions into pDONR-P2r-P3 (ThermoFisher, 12537023) for each of the six 3′-UTR sequences. We then performed LR reactions with pDONRP4P1r-p53RE containing the p53 response element (p53RE) from the *MDM2* promoter (ref.^6^; gift from the lab of G. Lahav), pDONR221-mCh containing mCherry coding sequence^37^ and a Gateway destination vector encoding the puromycin resistance gene to produce the expression plasmids that contain p53RE-mCherry-TRIAP1 3′-UTR, p53RE-mCherry-GADD45A 3′-UTR, p53RE-mCherry-DDB2 3′-UTR, p53RE-mCherry-TP53i3 3′-UTR, p53RE-mCherry-TP53i3 3′-UTR::1ARE or p53RE-mCherry-TP53i3 3′-UTR::2ARE. All BP and LR reaction products were verified by sequencing.

To develop the plasmids expressing pro-Caspase3, we first designed a gBlock (IDT) containing *CASP3* cDNA sequence flanked by *attB2* and *attB1* sites (Gateway cloning, ThermoFisher), then generated an entry clone of *CASP3* following the manufacturer’s Gateway cloning protocol for pDONR 221 (ThermoFisher). We then used LR reactions (Gateway cloning, ThermoFisher) with pDEST-Puro (G. Lahav) to generate plasmids containing p53RE-CASP3-TRIAP1 3′-UTR, p53RE-CASP3-GADD45A 3′-UTR and p53RE-CASP3-DDB2 3′-UTR. All reaction products were verified by sequencing.

### Human cell lines and culture

MCF-7 breast carcinoma cells were cultured in base growth medium of RPMI, 10% fetal bovine serum (FBS), 100 U/mL penicillin G, 100 mg/mL streptomycin sulfate, and 250 ng/mL amphotericin B. MCF-7 p53-Venus cells^7^ that express fluorescently tagged p53 were cultured in base growth medium containing 400 mg/mL neomycin.

To generate cell lines expressing constructs of p53RE-mCherry-DDB2 3′-UTR, p53RE-mCherry-TRIAP1 3′-UTR or p53RE-mCherry-GADD45A 3′-UTR, 2.5 μg of each construct was transfected into MCF-7 p53-Venus cells on separate 6-cm dishes using TransIT-LT1 Transfection Reagent (Mirus Bio, MIR 2300) and Opti-MEM I Reduced-Serum Medium (Gibco, 31985070) following the manufacturer’s protocol. The same procedure was used to transfect cell lines with p53RE-mCherry-TP53i3 3′-UTR, p53RE-mCherry-TP53i3 3′-UTR::1ARE or p53RE-mCherry-TP53i3 3′-UTR::2ARE. Two days after transfection, stable cells were selected and maintained using base growth medium containing 400 mg/mL neomycin and 0.5 μg/mL puromycin. Cell lines expressing p53RE-CASP3-DDB2 3′-UTR, p53RE-CASP3-TRIAP1 3′-UTR or p53RE-CASP3-GADD45A 3′-UTR were generated following the same procedure above to transfect the plasmids into MCF7 cells. Stable cells were selected and cultured in base growth medium containing 0.5 μg/mL puromycin after two days of transfection.

All cell lines were incubated and grown at 37 °C and 5% CO_2_.

### mRNA decay rate measurements

For MCF7 p53-Venus cell lines containing p53RE-mCherry-DDB2 3′-UTR, p53RE-mCherry-TRIAP1 3′-UTR or p53RE-mCherry-GADD45A, and for MCF7 cell lines with constructs of p53RE-CASP3-DDB2 3′-UTR, p53RE-CASP3-TRIAP1 3′-UTR or p53RE-CASP3-GADD45A 3′-UTR, 4 × 10^5^ cells were plated on two 6-cm dishes for each cell line and each time point of the experiment, two days prior to treatment. For p53-Venus cell lines containing p53RE-mCherry-TP53i3 3′-UTR, p53RE-mCherry-TP53i3 3′-UTR::1ARE or p53RE-mCherry-TP53i3 3′-UTR::2ARE, 4 × 10^5^ cells were plated on a 6-cm dish for each cell line and each time point of the experiment, two days prior to treatment. On the day of the treatment, cells were treated with 0.2 mM 5-ethynyl Uridine (EU) from the Click-iT Nascent RNA Capture Kit (ThermoFisher, C10365) and 400 ng/mL NCS (Sigma, N9162) for 3 hours, those of which were used for estimating newly synthesized RNA fraction and inducing double-strand breaks (DSB), respectively. Following 3 hours of treatment, cells were harvested by scraping at time points of 0, 1, 2 and 4 hours and frozen in a dry ice-ethanol bath. RNA of the samples was extracted using QIAshredder and RNeasy Kits (Qiagen), and RNA concentrations were measured by a UV spectrophometer to ensure that equal amount of 2.5 μg RNA was used to perform the Click-iT reaction of each sample, following the manufacturer’s protocol. RNA concentrations were measured again, so that 150 ng of each biotinylated RNA sample was used for the RNA binding reaction using the same kit and for the subsequent 20 μl cDNA synthesis reactions using an Invitrogen High Capacity cDNA Reverse Transcription Kit (ThermoFisher, 4374966). Samples and assays were put through thermal mixing (70 °C for 40 min, 60 °C for 30 s), hot start (95 °C for 1 min) and 35 cycles of PCR (96 °C for 5 s, 64 °C for 20 s), then melt curve acquisition at 64–95 °C with 0.5 °C resolution. The mRNA expression at the indicated time points for all samples was quantified in triplicate via qPCR with a CFX96 Real-Time PCR machine (Bio-Rad). Each qPCR reaction mixture contained 1ul of a sample, Maxima SYBR Green/Fluorescein Master Mix (Thermo Scientific, K0241), forward and reverse primers specific for the genes expressed, and water up to a 10 ul total reaction volume. For the constructs expressing *mCherry*, 5′-CACGAGTTCGAGATCGAGGG-3′ forward and 5′-CCCTTGGTCACCTTCAGCTT-3′ reverse primers were used; for those expressing *CASP3*, 5′-AAATACCAGTGGAGGCCGAC-3′ forward and 5′-ATGGCACAAAGCGACTGGAT-3′ reverse primers were used; and for *GAPDH* measurements, 5′-ACATCGCTCAGACACCATG-3′ forward and 5′-TGTAGTTGAGGTCAATGAAGGG-3′ reverse primers were used.

### Flow cytometry

Two days prior to treatment, 2 × 10^5^ MCF7 cells containing the constructs of p53RE-CASP3-DDB2 3′-UTR, p53RE-CASP3-TRIAP1 3′-UTR or p53RE-CASP3-GADD45A 3′-UTR were plated on 2 6-cm dishes each, and one set of plates was incubated with 400 ng/mL NCS 24 hours before the experiment. Cells were harvested by trypsinization and stained with 5 μL of the FITC annexin V dye and 10 μL of the 100 μg/mL PI dye following the manufacturer’s protocol (APC Annexin V Apoptosis Detection Kit with PI; BioLegend). Stained cells were analyzed by flow cytometry on a FacsCanto (BD Biosciences).

### Fluorescence expression measurements at single cell level

Time-lapse microscopy was used for the expression measurements of mCherry-tagged MCF7 p53-Venus cell lines. Two days prior to the microscopy experiment, cells were grown on poly-D-lysine-coated glass-bottom plates (MatTek Corporation) in selection medium. Approximately 45 mins before the experiment, medium of all samples were changed to transparent RPMI medium supplemented with 2% fetal bovine serum (FBS), 100 U/mL penicillin G, 100 mg/mL streptomycin sulfate, and 250 ng/mL amphotericin B. Cells were imaged on a Nikon Eclipse TiE inverted fluorescence microscope with a 20x plan apo objective (NA 0.75) using an iXon Ultra-888 camera (Andor) with constant temperature, CO_2_ concentration, and humidity maintained. Images were acquired every 20 min over a 24-hour period. The mCherry filter set contained filters of 540–580 nm for the excitation light, 585 nm for the dichroic beam splitter, and 593–668 nm for the emission light (Chroma). We analyzed images using NIS-Elements software (Nikon) and custom written ImageJ (NIH) and MATLAB software (Mathworks), which is available upon request.

## Supplementary information

Supplementary Figure S1

## References

[1] Tay S (2010). Single-cell NF-kappaB dynamics reveal digital activation and analogue information processing. Nature.

[2] Porter JR, Fisher BE, Batchelor E (2016). p53 Pulses Diversify Target Gene Expression Dynamics in an mRNA Half-Life-Dependent Manner and Delineate Co-regulated Target Gene Subnetworks. Cell Syst.

[3] Hafner A (2017). p53 pulses lead to distinct patterns of gene expression albeit similar DNA-binding dynamics. Nat Struct Mol Biol.

[4] Hansen AS, O’Shea EK (2013). Promoter decoding of transcription factor dynamics involves a trade-off between noise and control of gene expression. Mol Syst Biol.

[5] Lee RE, Walker SR, Savery K, Frank DA, Gaudet S (2014). Fold change of nuclear NF-kappaB determines TNF-induced transcription in single cells. Mol Cell.

[6] Lahav G (2004). Dynamics of the p53-Mdm2 feedback loop in individual cells. Nat Genet.

[7] Batchelor E, Mock CS, Bhan I, Loewer A, Lahav G (2008). Recurrent initiation: a mechanism for triggering p53 pulses in response to DNA damage. Mol Cell.

[8] Geva-Zatorsky N (2006). Oscillations and variability in the p53 system. Mol Syst Biol.

[9] Purvis JE (2012). p53 dynamics control cell fate. Science.

[10] Hoffmann A, Levchenko A, Scott ML, Baltimore D (2002). The IkappaB-NF-kappaB signaling module: temporal control and selective gene activation. Science.

[11] Ross J (1995). mRNA stability in mammalian cells. Microbiol Rev.

[12] Leppek K (2013). Roquin promotes constitutive mRNA decay via a conserved class of stem-loop recognition motifs. Cell.

[13] Chen CY, Shyu AB (1995). AU-rich elements: characterization and importance in mRNA degradation. Trends Biochem Sci.

[14] Grzybowska EA, Wilczynska A, Siedlecki JA (2001). Regulatory functions of 3′UTRs. Biochem Biophys Res Commun.

[15] Shiloh Y, Tabor E, Becker Y (1983). Abnormal response of ataxia-telangiectasia cells to agents that break the deoxyribose moiety of DNA via a targeted free radical mechanism. Carcinogenesis.

[16] Jao CY, Salic A (2008). Exploring RNA transcription and turnover *in vivo* by using click chemistry. Proc Natl Acad Sci USA.

[17] Janicke RU, Sprengart ML, Wati MR, Porter AG (1998). Caspase-3 is required for DNA fragmentation and morphological changes associated with apoptosis. J Biol Chem.

[18] Cameron DE, Bashor CJ, Collins JJ (2014). A brief history of synthetic biology. Nat Rev Microbiol.

[19] Cai L, Dalal CK, Elowitz MB (2008). Frequency-modulated nuclear localization bursts coordinate gene regulation. Nature.

[20] Nelson DE (2004). Oscillations in NF-kappaB signaling control the dynamics of gene expression. Science.

[21] Albeck JG, Mills GB, Brugge JS (2013). Frequency-modulated pulses of ERK activity transmit quantitative proliferation signals. Mol Cell.

[22] Levine JH, Lin Y, Elowitz MB (2013). Functional roles of pulsing in genetic circuits. Science.

[23] Locke JC, Young JW, Fontes M, Hernandez Jimenez MJ, Elowitz MB (2011). Stochastic pulse regulation in bacterial stress response. Science.

[24] Hao N, O’Shea EK (2011). Signal-dependent dynamics of transcription factor translocation controls gene expression. Nat Struct Mol Biol.

[25] Yissachar N (2013). Dynamic response diversity of NFAT isoforms in individual living cells. Mol Cell.

[26] Shankaran H (2009). Rapid and sustained nuclear-cytoplasmic ERK oscillations induced by epidermal growth factor. Mol Syst Biol.

[27] Shimojo H, Ohtsuka T, Kageyama R (2008). Oscillations in notch signaling regulate maintenance of neural progenitors. Neuron.

[28] Chen SH, Forrester W, Lahav G (2016). Schedule-dependent interaction between anticancer treatments. Science.

[29] Chen Xi, Chen Jia, Gan Siting, Guan Huaji, Zhou Yuan, Ouyang Qi, Shi Jue (2013). DNA damage strength modulates a bimodal switch of p53 dynamics for cell-fate control. BMC Biology.

[30] Elowitz MB, Leibler S (2000). A synthetic oscillatory network of transcriptional regulators. Nature.

[31] Stricker J (2008). A fast, robust and tunable synthetic gene oscillator. Nature.

[32] Golding, I., Paulsson, J., Zawilski, S. M. & Cox, E. C. Real-time kinetics of gene activity in individual bacteria. *Cell* **123** (2005). 10.1016/j.cell.2005.09.031 16360033

[33] Raj A, Peskin CS, Tranchina D, Vargas DY, Tyagi S (2006). Stochastic mRNA synthesis in mammalian cells. PLoS Biol.

[34] Raj A, van Oudenaarden A (2008). Nature, nurture, or chance: stochastic gene expression and its consequences. Cell.

[35] Suter D. M., Molina N., Gatfield D., Schneider K., Schibler U., Naef F. (2011). Mammalian Genes Are Transcribed with Widely Different Bursting Kinetics. Science.

[36] Chubb JR, Trcek T, Shenoy SM, Singer RH (2006). Transcriptional pulsing of a developmental gene. Curr Biol.

[37] Loewer, A., Batchelor, E., Gaglia, G. & Lahav, G. Basal dynamics of p53 reveal transcriptionally attenuated pulses in cycling cells. *Cell* **142** (2010). 10.1016/j.cell.2010.05.031 PMC3003696 20598361

