# Supplementary material for: Tuning of mRNA stability through altering 3′-UTR sequences generates distinct output expression in a synthetic circuit driven by p53 oscillations
Source: Sci Rep. 2019 Apr 12;9:5976. doi: 10.1038/s41598-019-42509-y (PMC6461691; doi:10.1038/s41598-019-42509-y)
Supplement: Supplementary file 1 — Supplementary Figure S1 [file 41598_2019_42509_MOESM1_ESM.pdf]

Supplementary Information for

"Tuning of mRNA stability through altering 3'-UTR sequences generates distinct output expression in a synthetic circuit driven by p53 oscillations"

Woo Seuk Koh, Joshua R. Porter, and Eric Batchelor

Included in this Supplementary Information is Supplementary Figure S1.

Supplementary Figure S1

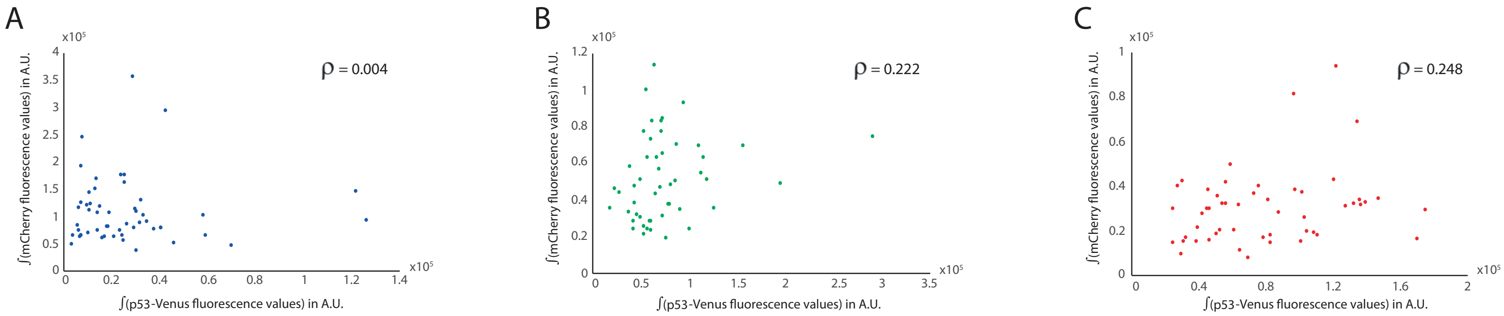

Supplementary Figure S1. Measurement of the correlation between oscillating p53 levels and mCherry output in single cells. Integrated levels of mean p53-Venus and mCherry fluorescence were calculated for the response in individual cells over 24 hours following double strand break induction with 400 ng/ml NCS. mCherry was expressed from a transcript containing the *DDB2* (A), *TRIAP1* (B), or *GADD45A* (C) 3'-UTR. Individual points represent integrated values in indvidual cells for approximately 50 cells per condition. Correlation was caluclated as Pearson's linear correlation ρ.
